# Thermodynamic investigation on the aqueous mixtures of choline chloride/propylene glycol deep eutectic solvent at T = (293.15 to 313.15) K

**DOI:** 10.1186/s13065-024-01153-y

**Published:** 2024-03-13

**Authors:** Aynaz Zarghampour, Parisa Jafari, Elaheh Rahimpour, Abolghasem Jouyban

**Affiliations:** 1 Pharmaceutical Analysis Research Center and Faculty of Pharmacy, Tabriz University of Medical Sciences, Tabriz, Iran; 2 Infectious and Tropical Diseases Research Center, Tabriz University of Medical Sciences, Tabriz, Iran

**Keywords:** Density, Deep eutectic solvent, Solute–solvent interactions, Excess molar volumes, Physicochemical properties

## Abstract

This work focuses on physicochemical features of the choline chloride/propylene glycol deep eutectic solvent (DES) + water mixtures by determining their density values in mono- and mixed- states over various temperatures in the range of 293.15–318.15 K. The density data obtained from measurements were utilized for the computation of various quantities such as excess molar volumes, molar volume, apparent molar volume, limiting apparent molar expansibility, and isobaric thermal expansion coefficient. Furthermore, the experimental densities were fitted to some mathematical equations such as Jouyban-Acree, Jouyban-Acreevan’t Hoff, modified Jouyban-Acree-van’t Hoff, Redlich–Kister and Emmerling. Studies of this nature can provide useful insights into solute–solvent interactions in aqueous solutions of DES, especially about to their novel application in drug solubilization.

**Supplementary Information:**

The online version contains supplementary material available at 10.1186/s13065-024-01153-y.

## Introduction

Deep eutectic solvents (DESs) as green solvents are an alternatives class for ionic liquids and are synthesized using a mixture of two or more organic substances. The combination of components in DESs results in a homogeneous mixture with a melting point that is lower than that of each component [1]. DESs are structured based on hydrogen bonding, where a hydrogen bond acceptor and hydrogen bond donor components interact with each other [2]. The main benefits of DESs are their biodegradability, cost-effectively, non-flammability and no further purification is required for their preparations [3, 4]. Moreover, DESs have a significant effect on the solute solubilization and enhance its aqueous solubility [5]. DESs are mostly based on choline chloride (ChCl), carboxylic acids, and other hydrogen bond donors *e.g.* succinic acid, urea, glycerol, and citric acid [6]. Before a solvent selection for any purpose, it needs to obtain some essential information on the targeted solvent including its volumetric features. Knowing these properties for DESs and their mixtures with water provides some helpful information on the solute–solvent interactions. Excess molar volume as a volumetric property demonstrates the non-ideality of a system and explains the intermolecular interactions [7–9].

Conducting such studies can yield useful insights regarding solute–solvent interactions in DESs' aqueous solutions, which have recently been employed for drug solubilization purposes. To date, there was some data on the thermodynamic behavior of an aqueous mixture of DESs including ChCl/ethylene glycol [10], ChCl /urea [11], ChCl/malonic acid [12], ChCl/phenol [13], ChCl/glycerol [14], ChCl/glucose [15], ChCl/oxalic acid [16], ChCl/triethylene glycol [17], halide salts/ethylene glycol [18], and amino acids/ lactic acid [19]. However, there is no data for the physicochemical properties of ChCl/propylene glycol (PG) at various temperatures in the literature. The goal of this work is to expand the database for physicochemical and thermodynamic properties of DESs + water mixtures at different temperatures for use in the solubilization procedures. It should be noted that the exact choice of solvent system will depend on various factors such as drug solubility, compatibility, and stability. The ChCl/PG + water system may not be suitable for all drugs and formulations, and alternative solvent systems may be required in some cases. However, regarding the comparison with other glycols, such as ethylene glycol and polyethylene glycols (PEGs), choosing the PG in the structure of DES has some advantages including: (i) ethylene glycol is toxic and not suitable for pharmaceutical applications. It is primarily used as an industrial solvent and antifreeze. Therefore, it is not a preferred choice for pharmaceutical formulations due to its potential health risks [20]. (ii) PEGs are polymers of ethylene glycol and are commonly used in pharmaceutical formulations as a solubilizing agent, stabilizer, and viscosity modifier [21]. However, compared to PG, PEGs may have higher viscosity and can be less efficient in solubilizing certain drugs. The specific ratio of 1 part ChCl to 3 parts PG was chosen for this study. The reason is that this composition has been reported as an effective DES for drug solubilization purposes in previous studies [22]. The DES system is formed by mixing ChCl and PG in a specific molar ratio, resulting in a lower melting point than either component alone. This lower melting point allows for the solubilization of drugs that are poorly soluble in traditional organic solvents. The 1:3 ChCl:PG ratio was selected as it provides a balance between solubilization efficiency and solvent viscosity, which is important for drug formulation and processing.

## Materials and methods

### Materials

This work utilized PG sourced from Scharlau Chemie (Spain), with a mass fraction purity greater than 0.995, ChCl obtained from Daejung (Korea) with a mass fraction purity greater than 0.999, and double-distilled water produced in the laboratory (Table 1).

**Table 1:** Some details of the purity and chemical structure of the employed materials^a^

Material	Mass fraction purity^a^	Source	Chemical formula	Molar mass/g mol^−1^	Structure
Propylene glycol (PG)	0.995	Scharlau Chemie	C_3_H_8_O_2_	76.09	
Choline chloride (ChCl)	> 0.999	Daejung, Korea	C_5_H_14_NClO	139.62	
Deionized water		Made in our laboratory	H_2_O	18.02	

^a^The purity of the employed chemicals was provided by the suppliers

### Preparation of ChCl/PG DES

Drying of ChCl was carried out in an oven at a temperature of 50 °C for 8 h. Subsequently, ChCl and PG were weighed in the desired amounts for preparing the solvent mixture, at a molar ratio of 1:3. The two components were then mixed and heated on a hot plate at a temperature of 80 ͦ C while stirring with a magnetic stirrer until a clear liquid was formed [23].

### Solvent mixtures preparation and determination of their densities

After DESs preparation, the eleven mixtures *i.e.* two mono- solvents and nine solvent mixtures in the DES ratio of 0.1–0.9 with a mass fraction interval of 0.1 were prepared and incubated at 293.2–313.2 K in a Nabziran Industrial Group incubator (Iran) until to reach temperature equilibrium. A 2 mL pycnometer was utilized for density measuring for each solution. Calibration of the pycnometer involves using the densities of distilled water at the respective temperatures used [24]. The pycnometer was filled with each prepared solution at desired temperatures and measured using a balance (model AB204-S, Mettler Toledo, Switzerland). With considering the weight of the empty pycnometer, the weight of the solvent mixture was obtained and used for density computing. The reported data were the mean of three replications at each temperature and composition.

## Results and discussion

### Density data and mathematical modeling

**Fig. 1:**
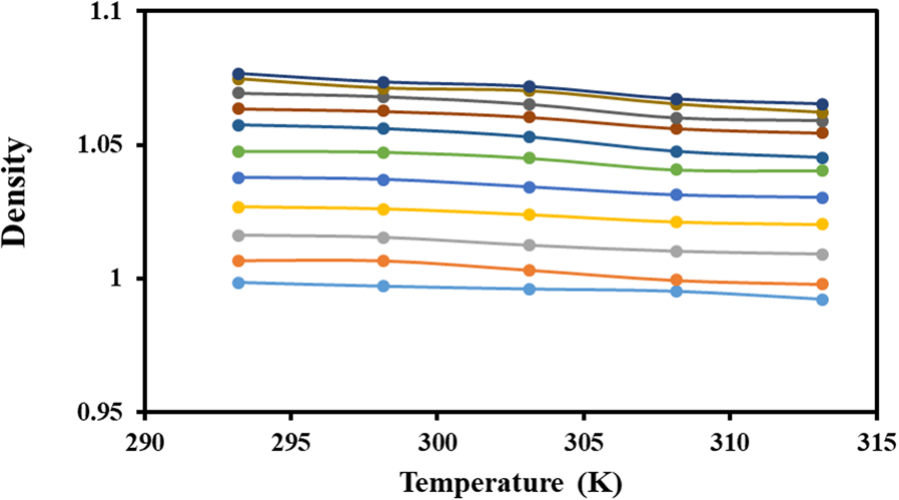
Density–temperature plot for a binary mixture of ChCl/PG DES and water

The measured densities (*ρ*_m_) at a temperature range of 293.15 to 313.15 K for the ChCl/PG DES and water mixtures in all compositions were given in Table 2. Figure 1 illustrated the density plot of ChCl/PG DES + water as a function of temperature, enabling clarity in visualization. Reliability of measured data using a pycnometer was investigated by repeating measurements using a density meter (Anton Paar DSA 5000 M). Density values for ChCl/PG DES + water mixtures at 298.2 K were determined using the density meter and results were given in electronic supplementary material (ESM) in Additional file 1:Table S1. As can be seen, there was no significant difference between the results of both measurements which confirmed the data provided by the pycnometer.

**Table 2:** The density data (g·cm^–3^) for a binary mixtures of ChCl/PG DES + water at different temperatures^a^

*x*_DES_^*b,c*^	293.2 K	298.2 K	303.2 K	308.2 K	313.2 K
0.0000	0.998 ± 0.000	0.997 ± 0.001	0.996 ± 0.000	0.994 ± 0.005	0.992 ± 0.000
0.0213	1.009 ± 0.001	1.007 ± 0.000	1.005 ± 0.000	1.003 ± 0.001	1.000 ± 0.000
0.0467	1.018 ± 0.001	1.016 ± 0.000	1.014 ± 0.000	1.011 ± 0.000	1.009 ± 0.001
0.0775	1.028 ± 0.000	1.025 ± 0.000	1.023 ± 0.000	1.020 ± 0.002	1.018 ± 0.000
0.1155	1.038 ± 0.000	1.035 ± 0.000	1.032 ± 0.0000	1.029 ± 0.001	1.026 ± 0.000
0.1638	1.047 ± 0.000	1.044 ± 0.000	1.041 ± 0.000	1.038 ± 0.000	1.035 ± 0.000
0.2271	1.056 ± 0.010	1.052 ± 0.002	1.050 ± 0.001	1.046 ± 0.000	1.043 ± 0.000
0.3137	1.064 ± 0.000	1.060 ± 0.000	1.058 ± 0.000	1.054 ± 0.002	1.051 ± 0.001
0.4394	1.071 ± 0.000	1.067 ± 0.001	1.0650 ± 0.000	1.061 ± 0.000	1.058 ± 0.002
0.6381	1.076 ± 0.002	1.072 ± 0.001	1.069 ± 0.001	1.065 ± 0.002	1.063 ± 0.001
1.0000	1.077 ± 0.003	1.073 ± 0.003	1.071 ± 0.000	1.067 ± 0.000	1.065 ± 0.001

^a^Standard uncertainty (*u*) for pressure, temperature, mole fraction of DESs and density is *u* (*P*) = 0.5 kPa, *u* (*T*) = 0.1 K, *u* (*x*_DES_) = 0.005, *u* (*ρ*_*m*_) = 0.05 kg m^−3^, respectively

^b^*x*_DES_ is the mole fraction of ChCl/PG DESs dissolved in water

^c^Standard uncertainty (*u*) for DES composition was estimated to be less than 0.05 mol ratio

Table 2 and Fig. 1 demonstrated that the densities rise with an increase in the mass fraction of DESs, while they decreased with an increase in temperature. The decrease in density can be attributed to the thermal expansion of the liquid volume with a temperature increase, leading to a less dense mixture at higher temperatures.

Equation (1) presents the temperature dependence of density values in the studied solutions, using a polynomial expression.

1 $$ \rho_{m} = a + bT + cT^{2} $$

Table 3 showed the model constants (*a, b*, and *c*) obtained from the least squares analysis for temperature T/K.

**Table 3:** The model constants (*a, b*, and *c*) from the least squares analysis at different temperatures for Eq. (1)

DES	*a*	10^5^. *b*	10^7^. *c*	*ARD*% ± 10^2^. *SD*
Bet/PG (molar ratio of 1:3)	1.123	− 1.759	− 8.663	2.15 ± 0.39

Furthermore, the corresponding molar volume was calculated using the density of neat DES with the following equation:

2 $$ V_{m,DES} = \frac{{M_{DES} }}{{\rho_{DES} }} $$

where $M_{DES}$ and $\rho_{DES}$ are the molar mass of DES and its density. The molar mass is computed by Eq. (3) [25].

3 $$ M_{DES} = \frac{{x_{HBA} M_{HBA} + x_{HBD} M_{HBD} }}{{x_{HBA} + x_{HBD} }} $$

where $x_{HBA}$, $x_{HBD}$, $M_{HBA}$ and $M_{HBD}$ are molar ratio and molar masses of ChCl as HBA and PG as HBD, respectively.

Table 4 showed that the variation of $V_{m}$ with temperature was comparable across the DES samples, with $V_{m}$ values exhibiting a slight increase as temperature increases.

**Table 4:** The molar masses and volumes of DES measured at various temperatures

DES	Molar mass / (g mol^−1^)	*T*/K				
		293.2	298.2	303.2	308.2	313.2
ChCl/PG (molar ratio of 1:3)	91.97	85.40	85.72	85.88	86.20	86.36

Jouyban-Acree [26, 27] (Eq. (4)), and Jouyban-Acree-van’t Hoff [28] (Eq. 5) models were used to represent the densities of pseudo-binary mixtures of ChCl/PG DES at different temperatures and each model constants together with the *ARD*s% and *SD*s were collected in Table 5.

4 $$ \ln \rho_{m} = x_{w} \ln \rho_{w} + x_{DES} \ln \rho_{DES} + x_{w} x_{DES} \sum\limits_{i = 0}^{N} {J_{i} \left( {\frac{{x_{w} - x_{DES} }}{T}} \right)^{i} } $$

5 $$ \ln \rho_{m} \,= w_{DES} \left( {A_{DES} + \frac{{B_{DES} }}{T}} \right) + w_{w} \left( {A_{w} + \frac{{B_{w} }}{T}} \right) + \frac{{w_{DES} w_{w} }}{T}\sum\limits_{i = 0}^{2} {J_{i} .(w_{DES} - w_{w} )^{i} } $$

in these equations, $\rho_{m}$, $\rho_{DES}$, $\rho_{w}$ are the density of mixture, neat DES and neat water, respectively. $x_{DES}$, $x_{w}$ correspond to the mole fraction of DES and water, respectively. $J_{i}$ is the model constants.

**Table 5:** Model constants and the *ARD*s% along with *SD* for the densities of pseudo-binary (ChCl/PG DES + water) mixtures

Jouyban-Acree [Eq. (4)]		Jouyban-Acree-van’t Hoff [Eq. (5)]	
*J*_0_ *J*_1_ *J*_2_ *ARD*% ± *SD*	9.905 7.794 5.101 0.05 ± 0.04	*A*_DES_ *B*_DES_ *A*_*w*_ *B*_*w*_ *J*_0_ *J*_1_ *J*_2_ *ARD*% ± *SD*	− 0.102 51.48 − 0.096 27.62 10.21 7.829 5.869 0.06 ± 0.03

To assess the precision of each model in depicting density values in the mixtures, the average relative deviation (*ARD*%) (Eq. 6) and standard deviation (*SD*) (Eq. 10) were calculated.

6 $$ ARD\% = \frac{100}{N}\sum {\left( {\frac{{\left| {\rho_{m}^{\exp } - \rho_{m}^{cal} \, } \right|}}{{\rho_{m}^{\exp } }}} \right)} $$

7 $$ SD = \sqrt {\frac{{\sum\limits_{i = 1}^{N} {\left| {\left( {\rho_{m}^{cal} - \overline{\rho }_{m}^{\exp } } \right)^{2} } \right|} }}{N}} $$

in Eqs. (6) and (7) $\rho_{m}^{\exp }$, $\overline{\rho }_{m}^{\exp }$, $\rho_{m}^{cal}$ and N are the experimental densities, the mean experimentally densities, the back-computed densities from different equations and the data point numbers, respectively.

In addition, modified Jouyban-Acree-van’t Hoff model [29, 30], Redlich–Kister [31] and Emmerling [32] were also employed for modeling the generated data and the related results were given in Additional file 1: Table S2.

### Excess molar volume and data correlation

To evaluate the non-ideality of (ChCl/PG DES + water) pseudo-binary mixtures, the $V^{E}$ values were calculated using Eq. (8).

8 $$ V^{E} = x_{DES} M_{DES} \left( {\frac{1}{{\rho_{m} }} - \frac{1}{{\rho_{DES} }}} \right) + x_{w} M_{w} \left( {\frac{1}{{\rho_{m} }} - \frac{1}{{\rho_{w} }}} \right) $$

Tables 6 reported the $V^{E}$ values found for the mixtures investigated. These values were fitted with Eq. (9) and the corresponding results were presented in Table 7. Additionally, Table 7 displayed the correlation outcomes of *V*_*m*_ values with Eq. (4).

9 $$ \ln \rho_{m} = x_{w} \ln \rho_{w} + x_{DES} \ln \rho_{DES} + x_{w} x_{DES} \sum\limits_{i = 0}^{N} {S_{i} \left( {x_{w} - x_{DES} } \right)^{i} } $$

**Table 6:** The dependence of $V^{E}$ values on DES mole fractions at operational temperatures for pseudo-binary mixtures of ChCl/PG DES (molar ratio of 1:3) in water

*x*_DES_	*T* = 293.2 K	*T* = 298.2 K	*T* = 303.2 K	*T* = 308.2 K	*T* = 313.2 K
	*V*^*E*^/cm^−3^ mol^−1^	*V*^*E*^/cm^−3^ mol^−1^	*V*^*E*^/cm^−3^ mol^−1^	*V*^*E*^/cm^−3^ mol^−1^	*V*^*E*^/cm^−3^ mol^−1^
0.0000	0.0000	0.0000	0.0000	0.0000	0.0000
0.0237	− 0.0627	− 0.0541	− 0.0438	− 0.0343	− 0.0269
0.0516	− 0.1057	− 0.0935	− 0.0818	− 0.0739	− 0.0623
0.0853	− 0.1628	− 0.1512	− 0.1378	− 0.1230	− 0.1133
0.1264	− 0.2293	− 0.2135	− 0.1982	− 0.1777	− 0.1627
0.1775	− 0.3028	− 0.2766	− 0.2587	− 0.2369	− 0.2124
0.2452	− 0.3624	− 0.3418	− 0.3207	− 0.2972	− 0.2764
0.3355	− 0.4389	− 0.4148	− 0.3967	− 0.3710	− 0.3514
0.4601	− 0.4865	− 0.4663	− 0.4431	− 0.4186	− 0.3957
0.6609	− 0.4105	− 0.3839	− 0.3694	− 0.3456	− 0.3283
1.0000	0.0000	0.0000	0.0000	0.0000	0.0000

a Standard uncertainties (*u*) are *u*(*T*) = 0.1 K, *u*(*p*) = 0.5 kPa, u(*x*_DES_) = 0.005 and the average combined expanded uncertainties *U*_c_ (level of confidence = 0.95, k = 2) is *U*_c_(*V*^E^) = 0.006 × 10^6^ m^3^ mol^−1^

**Table 7:** The model constants of Redlich–Kister obtained by fitting the $V^{E}$ values for the investigated solutions at different temperatures

Redlich–Kister equation [Eq. (7)]					
System	*T*/K	*S*_0_	*S*_1_	*S*_2_	*ARD*% ± 10^2^. *SD*
ChCl/PG DES + water	293.2	− 1.908	− 0.203	− 0.0219	3.54 ± 0.61
	298.2	− 1.820	− 0.231	0.1642	3.31 ± 0.55
	303.2	− 1.746	− 0.195	0.2531	2.59 ± 0.39
	308.2	− 1.650	− 0.188	0.3818	1.25 ± 0.32
	313.2	− 1.571	− 0.158	0.4747	3.35 ± 0.36
*OARD*% ± *SD*					2.81 ± 0.45

**Fig. 2:**
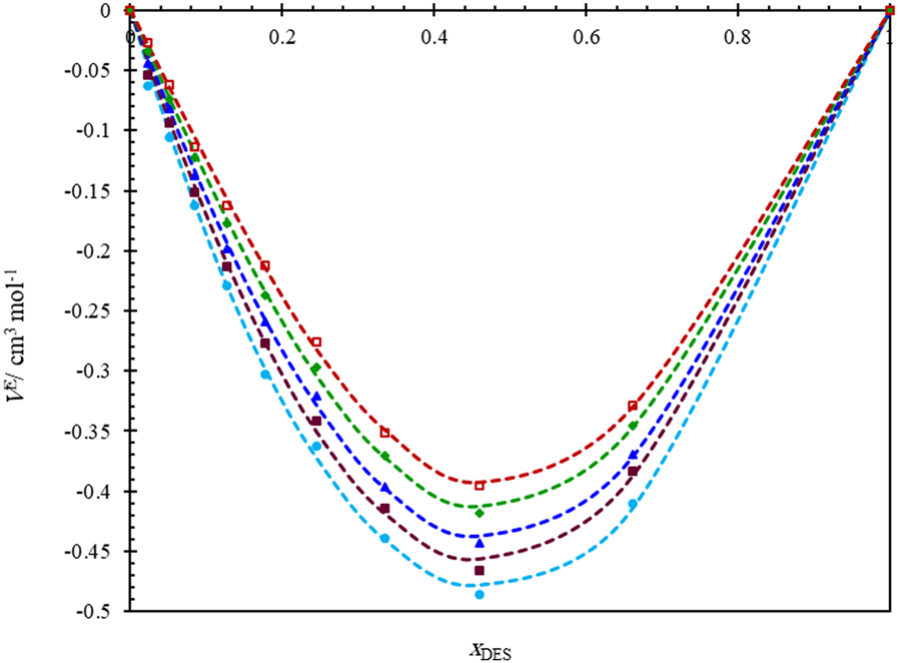
The excess molar volume (*V*^*E*^) for pseudo-binary mixtures of ChCl/PG DES (at a molar ratio of 1:3) in water calculated and plotted against DES mole fraction (*x*_*DES*_) at various temperatures: ( ), *T*/K = 293.15; ( ), *T*/K = 298.15; ( ), *T*/K = 303.15; ( ), *T*/K = 308.15; ( ), *T*/K = 313.15; the dashed lines obtained from fitting the *V*^E^ values with Redlich–Kister equation

Figure 2 displayed the experimentally and calculated $V^{E}$ values as a function of composition and temperature, allowing for a visual interpretation of their behavior. The negative trend observed throughout the range of composition and temperature indicated that the volume of mixtures was lower than that of an ideal mixture due to the strong interactions between DES and water molecules, likely attributed to hydrogen bonding between ChCl and PG with water molecules. This observation was consistent with previous findings for other pseudo-binary mixtures of (DES + water) [11–13, 19, 33, 34]. Additionally, the $V^{E}$ values became less negative at higher temperatures in the measured system, indicating that the intermolecular interactions between DES and water were weakened at elevated temperatures. This may result from an increase in hydrogen bond strength due to decreased intermolecular distances at lower temperatures. The behavior of the developed Redlich–Kister equation (Eq. 9) was represented by dashed lines.

### Apparent properties

The density values acquired for the aforementioned mixtures were utilized to calculate the apparent molar volume of DES ($V_{\varphi ,DES}$) in a water medium using the following equation:

10 $$ V_{\varphi ,DES} = \frac{{M_{DES} }}{{\rho_{m} }} - \frac{{\left( {\rho_{m} - \rho_{w} } \right)}}{{m_{DES} \rho_{m} \rho_{w} }} $$

The calculated values of ($V_{\varphi ,DES}$) were collected in Table 8. It was evident that the values of $V_{\varphi ,DES}$ were affected by the DES mole fraction and temperature. As both DES mole fraction and temperature increase, the values of $V_{\varphi ,DES}$ also increase. The dependency of $V_{\varphi ,DES}$ values on molality under isothermal conditions was fitted to the Redlich-Mayer equation [35, 36], which can be expressed as:

11 $$ V_{\varphi ,DES} = V_{\varphi ,DES}^{0} + A_{v} m_{DES}^{1/2} + B_{v} m_{DES} + C_{v} m_{DES}^{3/2} $$

**Table 8:** The calculated values of apparent molar volume of DESs ($V_{\varphi ,DES}$) and the corresponding partial molar volume ($\overline{V}_{DES}$) for the (ChCl/PG DES (molar ratio of 1:3) + water) mixtures at working temperatures^a,b^

System		293.15 K			298.15 K			303.15 K			308.15 K			313.15 K	
*m*_DES_	$$V_{\varphi ,DES} /$$$$(cm^{3} mol^{ - 1} )$$		$$\overline{V}_{DES} /$$$$(cm^{3} mol^{ - 1} )$$	$$V_{\varphi ,DES}/(cm^{3} mol^{ - 1} )$$		$$\overline{V}_{DES} /$$$$(cm^{3} mol^{ - 1} )$$	$$V_{\varphi ,DES} /$$$$(cm^{3} mol^{ - 1} )$$		$$\overline{V}_{DES} /$$$$(cm^{3} mol^{ - 1} )$$	$$V_{\varphi ,DES} /$$$$(cm^{3} mol^{ - 1} )$$		$$\overline{V}_{DES} /$$$$(cm^{3} mol^{ - 1} )$$	$$V_{\varphi ,DES} /$$$$(cm^{3} mol^{ - 1} )$$		$$\overline{V}_{DES} /$$$$(cm^{3} mol^{ - 1} )$$
0.0213	82.46		82.81	83.18		83.39	83.82		83.92	84.59		84.60	85.10		85.04
0.0467	83.13		83.59	83.71		83.83	84.12		84.18	84.61		84.63	85.02		85.00
0.0775	83.29		83.80	83.76		83.84	84.09		84.14	84.61		84.63	84.89		84.89
0.1155	83.41		83.93	83.87		83.92	84.16		84.20	84.66		84.68	84.95		84.96
0.1638	83.55		84.06	84.03		84.07	84.30		84.32	84.75		84.77	85.06		85.07
0.2271	83.80		84.27	84.21		84.24	84.46		84.48	84.89		84.91	85.14		85.16
0.3137	84.00		84.39	84.39		84.41	84.61		84.63	85.01		85.03	85.24		85.25
0.4394	84.29		84.65	84.65		84.66	84.87		84.88	85.24		85.26	85.46		85.47
0.6381	84.75		86.13	85.11		85.12	85.30		85.30	85.66		85.66	85.84		85.84

^a^Standard uncertainty (*u*) for pressure, temperature, mole fraction of DESs and density is *u* (*P*) = 0.5 kPa, *u* (*T*) = 0.1 K and *u* (*m*_DES_) = 0.005 mol kg^−1^, respectively

^b^Standard uncertainty (*u*) for DESs composition was estimated to be less than 0.05 mol ratios

Equation (11) expresses $V_{\varphi ,DES}^{0} ({\text{cm}}^{{3}} {\text{ mol}}^{{ - 1}} )$ and $A_{\upsilon } ({\text{cm}}^{{3}} {\text{ kg}}^{{{1}/{2}}} {\text{ mol}}^{{ - {3}/{2}}} )$ as the apparent molar volume in infinite dilution and the Debye–Huckel limiting slopes for the apparent molar volume, respectively. In addition,.$B_{\upsilon } ({\text{cm}}^{{3}} {\text{ kg mol}}^{{ - {2}}} )$ and $C_{\upsilon } ({\text{cm}}^{{3}} {\text{ kg}}^{{{3}/{2}}} {\text{ mol}}^{{ - {5}/{2}}} )$ are considered as empirical constants that depend on the solute, solvent, and temperature.

$m_{DES}$ represents the molality of DES present in the mixtures. The parameters of Eq. (11) were calculated for DES in its aqueous pseudo-binary mixtures using the least square fit method, and the outcomes were depicted in Table 9. According to the presented results in Table 9, it was evident that the values for DES in water were positive, indicating the existence of robust interactions between DES and water.

**Table 9:** Limiting apparent molar volume of DES ($V_{\varphi ,DES}^{0}$), adjustable parameters of Eq. (11) (*A*_V_, *B*_v_ and *C*_V_), along with *SD*, empirical parameters of Eq. (15) (a, b, and c), partial molar expansibility ($\phi_{E,DES}^{0}$), isobaric thermal expansion ($\alpha_{p,m}$) and Hepler’s constant [$\left( {\frac{{\partial^{2} V_{\varphi ,DES}^{0} }}{{\partial T^{2} }}} \right)_{p}$] for the pseudo-binary (hCl/PG DES + water) mixtures at *T* = (293.15–313.15) K

*T*/K	$V_{\varphi ,DES}^{0}$/(cm^3^ mol^−1^)	$A_{v}$/(cm^3^ mol^−3/2^ kg^1/2^)	$B_{v}$/(cm^3^ mol^−2^ kg)	10^3^.$C_{v}$/(cm^3^ mol^−5/2^ kg^3/2^)	*ARD*% ± *SD* ($V_{\varphi ,DES}^{0}$/(cm^3^ mol^−1^))	*a*_*1*_/(cm^3^ mol^−1^)	*b*_*1*_/(cm^3^ mol^−1^ K^−1^)	*c*_*1*_/(cm^3^ mol^−1^ K^−2^)	$\phi_{E,DES}^{0}$/(cm^3^ mol^−1^ K^−1^)	$\alpha_{p,m}$/(K^−1^)	$\left( {\frac{{\partial^{2} V_{\varphi ,DES}^{0} }}{{\partial T^{2} }}} \right)_{p}$
ChCl/PG DES (molar ratio of 1:3) + water											
293.2	81.78	0.8806	− 0.1146	5.669	0.09 ± 0.10	− 90.837	0.974	− 0.0010	0.203	2.482	0.771
298.2	82.71	0.6131	− 0.0726	3.545	0.06 ± 0.08				0.190	2.297	0.784
303.2	83.65	0.2243	− 0.0075	1.232	0.04 ± 0.05				0.177	2.116	0.797
308.2	84.57	− 0.053	0.0380	− 2.183	0.02 ± 0.02				0.164	1.939	0.810
313.2	85.28	− 0.2780	0.0730	− 3.973	< 0.01 ± 0.04				0.151	1.771	0.823

Alternatively, the rise in $V_{\varphi ,DES}^{0}$ values at higher temperatures can be explained by examining the primary and secondary solvation layers. An augmentation in temperature results in the release of solvent molecules from the secondary hydration layer of solutes into the bulk of the solvent, leading to the expansion of the primary layer. This was reported in recent studies for numerous systems [19, 37, 38]. In comparison to the $B_{\upsilon }$ values, the higher $V_{\varphi ,DES}^{0}$ values suggest that the interactions between DES and water were stronger than those of the self-interactions of molecules (such as DES-DES or water-water) across all temperatures.

Apparent molar volume was utilized to determine the partial molar volume ($\overline{V}_{DES}$) of ChCl/PG DES in water medium by the following equation [39]:

12 $$ \overline{V}_{DES} = V_{\varphi ,DES} + m_{DES} \left( {\frac{{\partial V_{\varphi ,DES} }}{{\partial m_{DES} }}} \right) $$

Table 8 provided the computed $\overline{V}_{DES}$ values, which exhibit the same pattern as the $V_{\varphi ,DES}$ values.

The temperature-dependence of $V_{\varphi ,DES}^{0}$ values can be expressed with Eq. (13) [19], as seen Table 9.

13 $$ V_{\varphi ,DES} = a_{1} + b_{1} T + c_{1} T^{2} $$

Two crucial parameters were calculated using the first and second derivatives of $V_{\varphi ,DES}^{0}$ values with regard to temperature, *i.e.* the limiting apparent molar expansibility ($\phi_{E,DES}^{0}$) of neat DES and Hepler’s constant ($\left( {\frac{{\partial^{2} V_{\varphi ,DES}^{0} }}{{\partial T^{2} }}} \right)_{p}$) with the Eqs. (14) and (15). The results were also reported in Table 9.

14 $$ \phi_{E,DES}^{0} = \left( {\frac{{\partial V_{\varphi ,DES}^{0} }}{\partial T}} \right)_{p} = B + 2CT $$

15 $$ \left( {\frac{{\partial^{2} V_{\varphi ,DES}^{0} }}{{\partial T^{2} }}} \right)_{p} = 2C $$

Table 9 displayed the favorable $\phi_{E,DES}^{0}$ values for ChCl/PG DES aqueous pseudo-binary solutions at the operating temperature, which indicate solvation and electrostriction of solutes in an aqueous environment [40]. This observation could be explained by the speedy transfer of water molecules from the DES hydration layers to the neat water. Additionally, the positive values of Hepler's constant revealed that ChCl/PG DES has a structure-making effect on the water medium at the operating temperatures, as mentioned in reference [41].

Understanding the impact of temperature on liquid expansion is crucial for equipment design. As the temperature of a liquid increases, its volume also increases due to the kinetic energy of its molecules. This phenomenon is known as thermal expansion. The amount of expansion is proportional to the temperature change and the initial volume of the liquid. This relationship is expressed by the coefficient of thermal expansion, which is a measure of how much a material expands per unit length for a given temperature change. This information can be obtained through the isobaric thermal expansion coefficient ($\alpha_{p,m}$) calculated using Eq. (16).

16 $$ \alpha_{p,m} = \frac{{\phi_{E,DES}^{0} }}{{V_{\varphi ,DES}^{0} }} $$

Table 9 indicates that the $\alpha_{p,m}$ values declined as the temperature increased.

## Conclusion

The densities of ChCl and PG (at a ratio of 1:3) deep eutectic solvents were experimentally determined over a range of temperatures, and based on these measurements, certain physicochemical properties such as $V^{E}$, $\phi_{E,DES}^{0}$, $\phi_{E,DES}^{0}$, $\alpha_{p,m}$, and $\left( {\frac{{\partial^{2} V_{\varphi }^{0} }}{{\partial T^{2} }}} \right)_{p}$ were calculated. The fact that $V^{E}$ values were negative for the entire range of mole fractions and temperatures in the aqueous pseudo-binary DES mixtures suggests that DES and water molecules interact favorably with each other. Additionally, an increase in the temperature resulted in an increase in the limiting apparent molar volume ($V_{\varphi ,DES}^{0}$), which further supports the idea that favorable DES-water interactions occur in these mixtures. In addition, the fact that Helper’s constant had positive values indicates that DES functions as a structure-maker in a water-based medium. Furthermore, several models, including Jouyban-Acree, Jouyban-Acree-van’t Hoff, modified Jouyban-Acree-van’t Hoff, Redlich–Kister, and Emmerling were utilized to represent the experimental densities. Based on the results, the order of the models’ effectiveness in density correlation was determined as follows: Redlich–Kister equation (0.01 ± 0.01) > Emmerling model (0.02 ± 0.02) > Jouyban-Acree (0.05 ± 0.04) = modified Jouyban-Acree-van’t Hoff model (0.05 ± 0.04) > Jouyban-Acree-van’t Hoff model (0.06 ± 0.03).

### Supplementary Information

**Additional file 1: Table S1.** The density data (g·cm–3) for binary mixtures of ChCl/PG DES + water at 298.2 K using pycnometer and density meter. **Table S2.** Model constants and the* ARD*s% along with the* SD* for the densities of pseudo-binary (ChCl/PG DES + water) mixtures.

## Data Availability

The datasets used and/or analysed during the current study are available from the corresponding author on reasonable request.
