# Supplementary material for: Thermodynamic investigation on the aqueous mixtures of choline chloride/propylene glycol deep eutectic solvent at T = (293.15 to 313.15) K
Source: BMC Chem. 2024 Mar 13;18(1):51. doi: 10.1186/s13065-024-01153-y (PMC10935834; doi:10.1186/s13065-024-01153-y)
Supplement: Supplementary file 1 — Additional file 1: Table S1. The density data (g·cm–3) for binary mixtures of ChCl/PG DES + water at 298.2 K using pycnometer and density meter. Table S2. Model constants and the ARDs% along with the SD for the densities of pseudo-binary (ChCl/PG DES + water) mixtures. [file 13065_2024_1153_MOESM1_ESM.docx]

**Electronic supplementary material**

**Thermodynamic investigation on the aqueous mixtures of choline chloride/propylene glycol deep eutectic solvent at T = (293.15 to 313.15) K**

**Aynaz Zarghampour ^a,b^, Parisa Jafari ^b^, Elaheh Rahimpour ^b,c^,[[1]](#footnote-1), Abolghasem Jouyban ^b^**

^a^ *Student Research Committee, Tabriz University of Medical Sciences, Tabriz, Iran*

^b^ *Pharmaceutical Analysis Research Center and Faculty of Pharmacy, Tabriz University of Medical Sciences, Tabriz, Iran*

*^c^ Infectious and Tropical Diseases Research Center, Tabriz University of Medical Sciences, Tabriz, Iran.*

**Table S1**

The density data (g·cm^–3^) for binary mixtures of ChCl/PG DES + water at 298.2 K using pycnometer and density meter

| *x*_DES_ | Density meter | Pycnometer |
| --- | --- | --- |
| 0.0000 | 0.995 | 0.997 |
| 0.0213 | 1.008 | 1.007 |
| 0.0467 | 1.015 | 1.016 |
| 0.0775 | 1.026 | 1.025 |
| 0.1155 | 1.033 | 1.035 |
| 0.1638 | 1.044 | 1.044 |
| 0.2271 | 1.053 | 1.052 |
| 0.3137 | 1.061 | 1.060 |
| 0.4394 | 1.064 | 1.067 |
| 0.6381 | 1.073 | 1.072 |
| 1.0000 | 1.070 | 1.073 |

The modified Jouyban-Acree-van’t Hoff model (Eq. (1)), Redlich-Kister (Eq. (2)) and Emmerling (Eq. (3)) models were used for density data modeling and the results were given in Table S2.

$\ln\rho_{m}=D_{1}+\frac{D_{2}}{T}+D_{3}w_{DES}+D_{4}\frac{w_{DES}}{T}+D_{5}\frac{w_{_{DES}}^{2}}{T}+D_{6}\frac{w_{_{DES}}^{3}}{T}+D_{7}\frac{w_{_{DES}}^{4}}{T}$ (1)

$\ln\rho_{m}=x_{w}\ln\rho_{w}+x_{DES}\ln\rho_{DES}+x_{w}x_{DES}\sum_{i=0}^{N} S_{i}\left(x_{w}-x_{DES}\right)^{i}$ (2)

$\rho_{m}=x_{w}\rho_{w}+x_{DES}\rho_{DES}+x_{w}x_{DES}\left[\begin{matrix} p_{1}+p_{2}T+p_{3}T^{2}+\left(x_{w}-x_{DES}\right)\left(p_{4}+p_{5}T+p_{6}T^{2}\right)+ \\ \left(x_{w}-x_{DES}\right)^{2}\left(p_{7}+p_{8}T+p_{9}T^{2}\right) \end{matrix}\right]$ (3)

in these equations, $\rho_{m}$, $\rho_{DES}$, $\rho_{w}$ are the density of mixture, neat DES and neat water, respectively. $x_{DES}$, $x_{w}$ correspond to the mole fraction of DES and water, respectively. *A*_DES_, *B*_DES_, *A*_w_, *B*_w_, *D*_i_, $S_{i}$, and $P_{i}$ are the model constants. To assess the precision of each model in depicting density values in the mixtures, the average relative deviation (ARD%) and standard deviation (SD) (Eq. were calculated.

**Table S2.** Model constants and the *ARD*s% along with the *SD* for the densities of pseudo-binary (ChCl/PG DES + water) mixtures.

| **Redlich-Kister** | | | | | | | | |
| --- | --- | --- | --- | --- | --- | --- | --- | --- |
| ***T*/K** | | ***S*_0_** | ***S*_1_** | | | ***S*_2_** | ***ARD*% ± *SD*** | |
| **293.2** | | 0.038 | 0.025 | | | 0.020 | 0.01 ± 0.01 | |
| **298.2** | | 0.035 | 0.025 | | | 0.019 | 0.01 ± 0.01 | |
| **303.2** | | 0.033 | 0.026 | | | 0.016 | 0.01 ± 0.01 | |
| **308.2** | | 0.030 | 0.026 | | | 0.015 | 0.01 ± 0.01 | |
| **313.2** | | 0.027 | 0.026 | | | 0.014 | 0.01 ± 0.01 | |
| ***Overall ARD*% (*OARD*%) ± *SD*** | |  |  | | |  | 0.01 ± 0.01 | |
| **The modified Jouyban-Acree-van’t Hoff** | | | | **Emmerling expression** | | | |
| ***D*_1_**  ***D*_2_**  ***D*_3_**  ***D*_4_**  ***D*_5_**  ***D*_6_**  ***D*_7_**  ***ARD*% ± *SD*** | -0.1210  35.32  0.0001  24.18  13.27  -15.45  NS *^a^*  0.05 ± 0.04 | | | ***D*_1_**  ***D*_2_**  ***D*_3_**  ***D*_4_**  ***D*_5_**  ***D*_6_**  ***D*_7_**  ***D*_8_**  ***D*_9_**  ***ARD*% ± *SD*** | 0.196  -0.001  NS *^a^*  0.028  NS *^a^*  NS *^a^*  0.073  NS *^a^*  -5.909 × 10^-7^  0.02 ± 0.02 | | | | |

*^a^* NS denotes to not statistically significant (p-value > 0.05).

1. Corresponding author. E-mail: rahimpour_e@yahoo.com [↑](#footnote-ref-1)
